# Bilateral Elongated Mandibular Coronoid Process and Restricted Mouth Opening: A Case Report

**DOI:** 10.2174/1874210601711010670

**Published:** 2017-12-27

**Authors:** Thomas Starch-Jensen, Annette Dalgaard Kjellerup

**Affiliations:** Department of Oral and Maxillofacial Surgery, Aalborg University Hospital, Aalborg, Denmark

**Keywords:** Dentistry, Diagnostic imaging, Facial bones, General surgery, Hyperplasia, Temporomandibular joint

## Abstract

**Introduction:**

Mandibular coronoid process hyperplasia is an uncommon congenital or developmental temporomandibular joint disorder, characterized by elongation of the coronoid process, which may cause limited mandibular movement as a consequence of interference between the hyperplastic coronoid process and the medial surface of the zygomatic arch.

**Methods:**

Mandibular coronoid process hyperplasia commonly affects males in the second decade of life and the exact aetiology and pathogenesis is unknown. The condition can be uni- or bilateral. Progressive painless reduction in mouth opening is the main clinical finding and computed tomography is the most reliable imaging modality for confirming the diagnosis.

**Results:**

Surgical intervention involving coronoidectomy and long-term intensive postoperative physiotherapy is the treatment of choice for mandibular coronoid process hyperplasia with impingement on the zygomatic bone and limited mouth opening. However, surgically induced fibrosis and the tendency for mandibular coronoid process regrowth may cause relapse and renewed limited mouth opening. Vigorous physical therapy should therefore be initiated shortly after surgery.

**Conclusion:**

The purpose of this case report is to present the clinical and radiographic features of elongated mandibular coronoid process in an 18-year-old male with limited mouth opening, and to discuss the various surgical treatment modalities.

## 1 INTRODUCTION

Mandibular coronoid process hyperplasia (MCPH) is classified as a congenital or developmental temporo-mandibular joint disorder and defined as an abnormal elongation of the coronoid process consisting of histologically normal bone [1-3]. MCPH is a rare condition which may enlarge the coronoid processes to such an extent that they can impinge upon the medial surfaces of the zygomatic arches and causes mouth opening limitation [1, 3]. MCPH was first described by Langenbeck in 1853 and the first case of mandibular hypomobility by coronoid process enlargement was reported in 1899 [4, 5]. The etiology of MCPH has not been established, but several explanations have been suggested such as trauma, increased activity of the temporal muscles, hormonal or genetic components, and temporomandibular disorders [1]. MCPH mostly affects males in the second decade of life [1, 3]. MCPH might be unilateral or bilateral, but the bilateral form occurs more frequently [1]. The unilateral form is often seen in females, whereas the bilateral form is more common in males [1]. The main clinical symptom of elongated mandibular coronoid process impinging upon the zygomatic bone is a progressive painless reduction in mouth opening [1, 3, 6]. On conventional radiograph imaging, MCPH usually presents as a hyperplastic coronoid process projecting into the infratemporal fossa. Computed tomography is mandatory for accurately evaluation of the association between the hyperplastic coronoid process and the zygomatic bone, and thus plays an important role in diagnosing and planning the surgical treatment [7, 8]. Coronoidectomy using an intraoral approach followed by vigorous long-term postoperative physiotherapy is the recommended treatment modality for MCPH with impingement on the zygomatic bone and limited mouth opening [6, 9-12]. However, surgically induced fibrosis and the tendency for mandibular coronoid process regrowth may cause relapse and renewed limited mouth opening [3]. Vigorous physical therapy should therefore be initiated in the early postoperative phase and patients should be monitored for possible regrowth of the coronoid process. Coronoidotomy or wedge subcoronoid ostectomy are alternative surgical treatment modalities for MCPH and the treatment choice should be based on a careful evaluation of the individual case [13, 14].

The purpose of presenting this case report is to summarize the current knowledge about this uncommon congenital or developmental temporomandibular joint disorder and emphasize the association between MPCH and limited mouth opening.

## 2 CASE PRESENTATION

An 18-years old male was referred by his general dental practitioner to the Department of Oral and Maxillofacial Surgery, Aalborg University Hospital, Denmark, due to a progressive asymptomatic impairment of mouth opening interfering with eating, speaking, and maintaining oral hygiene. The patient´s medical history was unremarkable and there was no history of facial trauma or surgery. The patient had previously undergone orthodontic treatment.

**Fig. (1):**
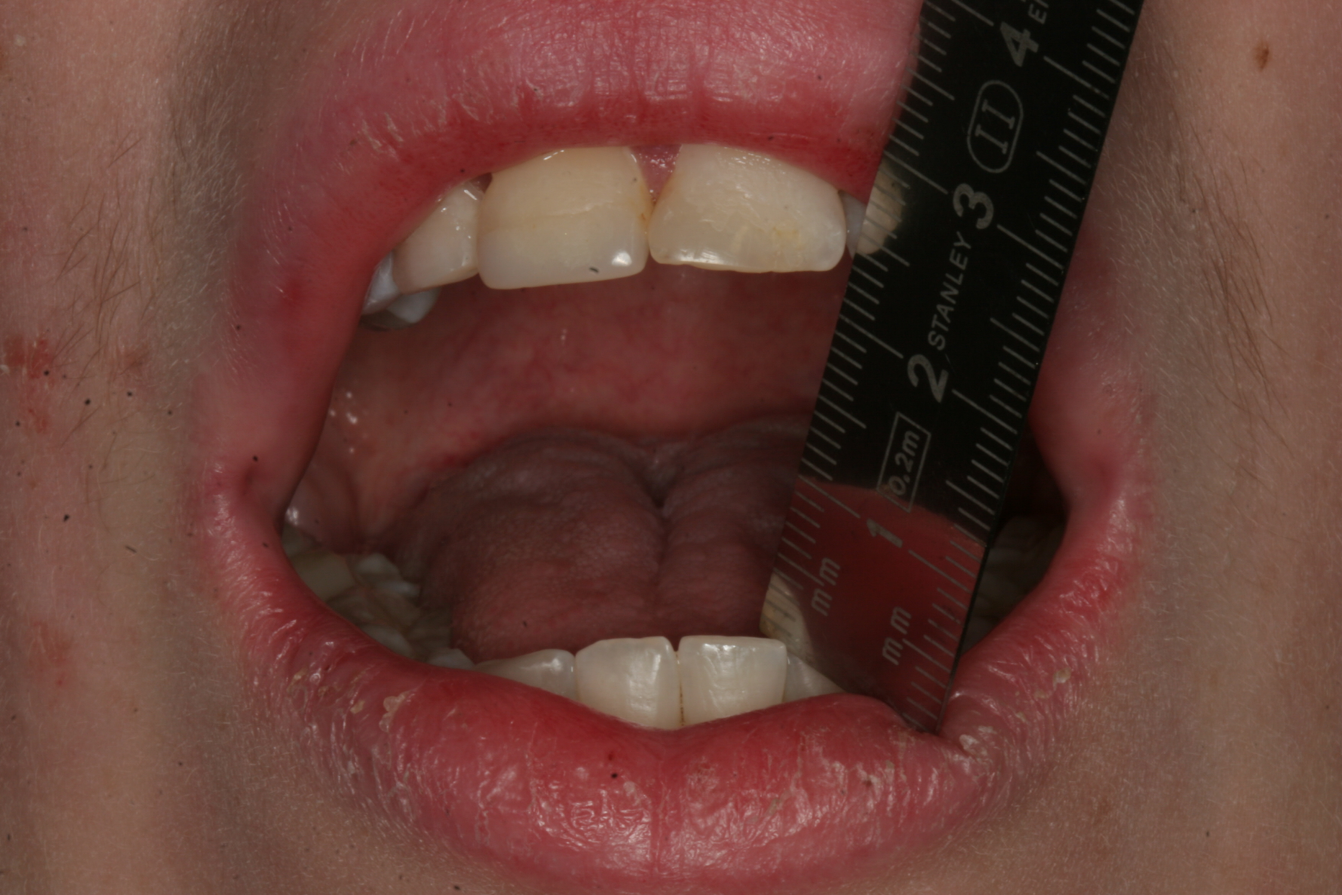
Preoperative maximal interincisal opening of 22 mm.

**Fig. (2):**
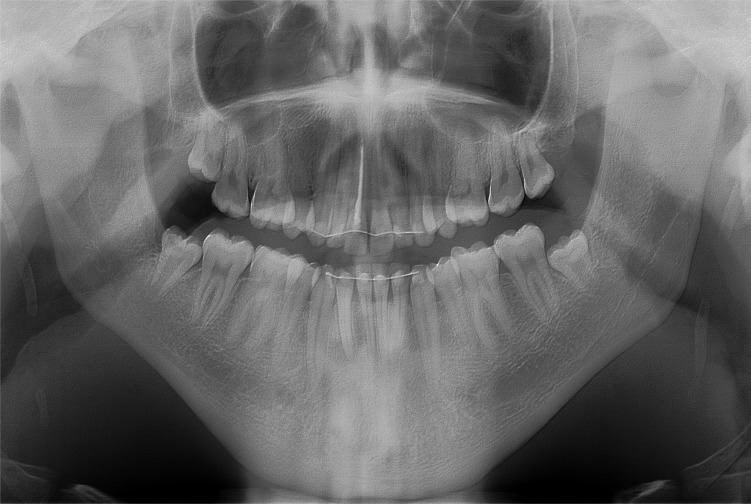
Preoperative orthopantomogram showing the elongated mandibular coronoid process projecting into the infratemporal fossa.

**Fig. (3):**
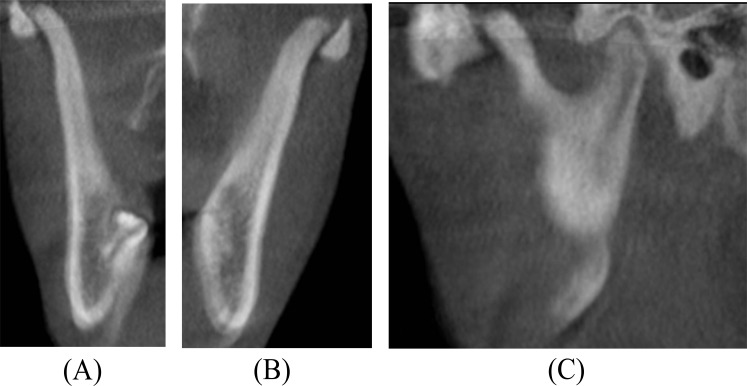
Preoperative Cone Beam Computed Tomography scan. (A) Coronal image showing close approximation of the right coronoid process to the medial surface of the zygomatic bone. (B) Coronal image showing close approximation of the left coronoid process to the medial surface of the zygomatic bone (C) Sagittal image showing enlargement of the right coronoid process impinged on the zygomatic bone.

On physical examination, the temporalis and masseter muscles were slightly tender on palpation, but no masticatory muscle pain and temporomandibular joint pain or sounds were observed. The maximal interincisal mouth opening was 22 mm with lateral movements to the right and left of 5 mm and 6 mm, respectively (Fig. **1**). Attempts to increase mouth opening by manipulation was impossible. Intraoral clinical examination revealed intact dental arches and an Angle class I occlusion. A panoramic radiography showed bilateral enlargement of the mandibular coronoid process projecting into the infratemporal fossa (Fig. **2**). Cone beam computed tomography with three-dimensional recons-truction revealed bilateral elongated mandibular coronoid processes impinging upon the zygomatic bone during mouth opening (Fig. **3**). On the basis of the clinical and radiographic findings, a working diagnosis of MCPH was made. Due to the restricted mouth opening and impingement of the coronoid processes with the zygomatic arches, the decision was made to perform bilateral coronoidectomy.

**Fig. (4):**
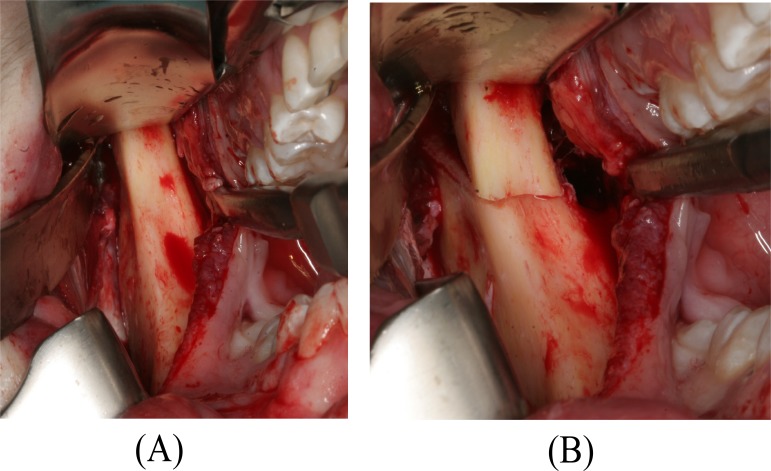
Intraoperative clinical photo. (A) The elongated mandibular coronoid process exposed by an intraoral approach. (B) A low coronoidectomy performed with reciprocating saw.

**Fig. (5):**
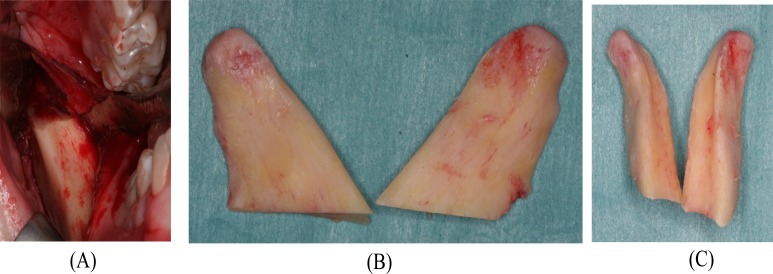
Intraoperative clinical photo. (A) The mandibular coronoid processes after coronoidectomy. (B) The resected coronoid processes. (C) Frontal image of the resected coronoid processes.

**Fig. (6):**
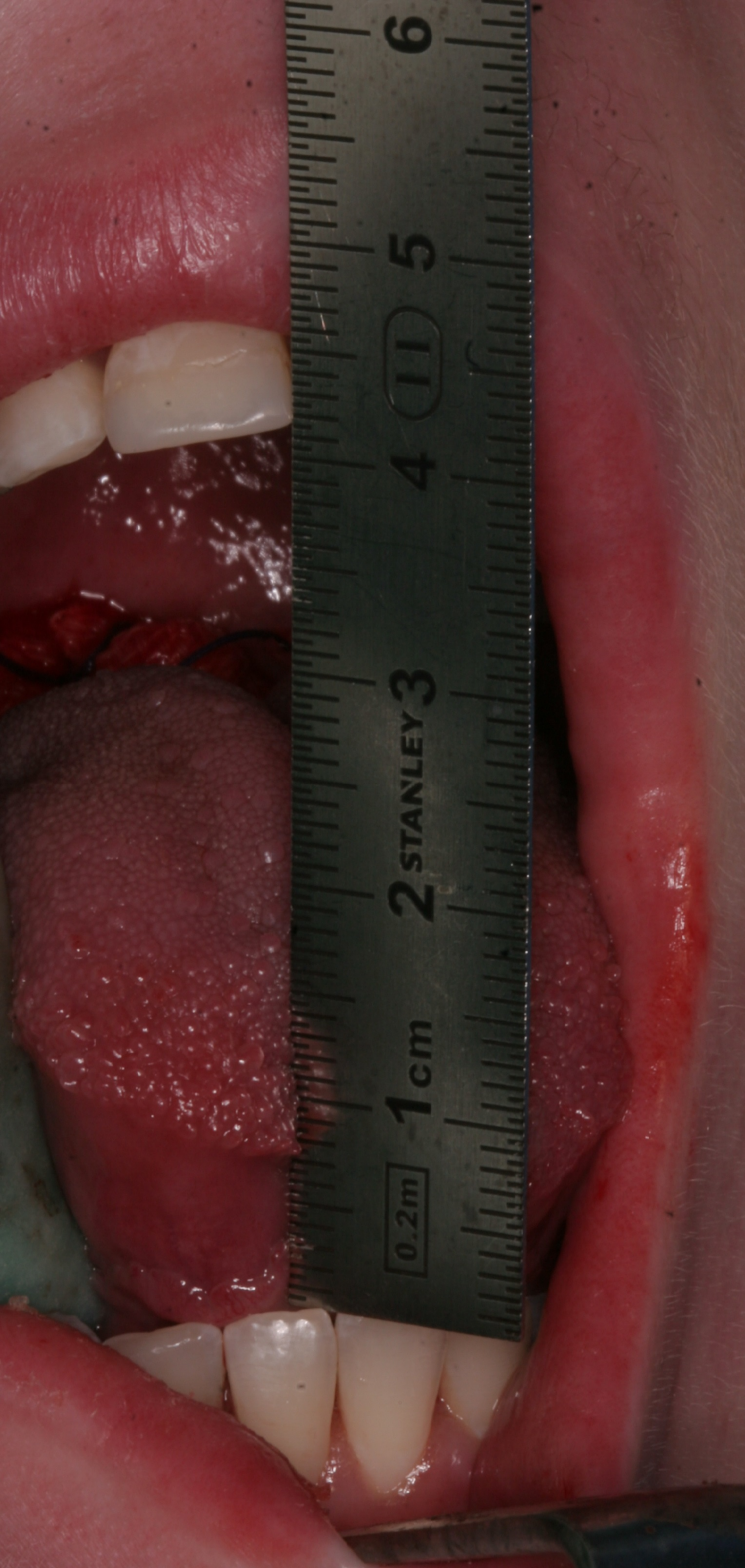
The interincisal mouth opening increased to 42 mm in the operation room.

The surgical procedure was conducted in general anaesthesia with nasotracheal intubation, supplemented by local anaesthesia. An intraoral incision, similar to the surgical approach for a sagittal split osteotomy, provided the necessary access to the coronoid processes. The periosteum and the masseter muscle were elevated over the ascending ramus. The insertion of the temporal muscles was released of the temporalis attachment on the anterior border of the mandibular ramus and the tendon attachment was cut from the coronoid process. A channel retractor was placed into the sigmoid notch and a ramus clamp was secured over the coronoid process before a low coronoidectomy was performed from the sigmoid notch to the anterior oblique ridge with a reciprocating saw (Fig. **4**). The coronoid process on both sides was removed with forceps and surgical recontouring of the mandibular ramus was performed with a drill (Fig. **5**). The wound was irrigated with saline and closed with resorbable continuous sutures. The interincisal mouth opening increased to 42 mm in the operation room (Fig. **6**). The patient was discharged later the same day. Healing was uneventful. Histopathologic examination of the resected coronoid process revealed normal bone, confirming the diagnosis of primary bony hyperplasia of the coronoid processes.

**Fig. (7):**
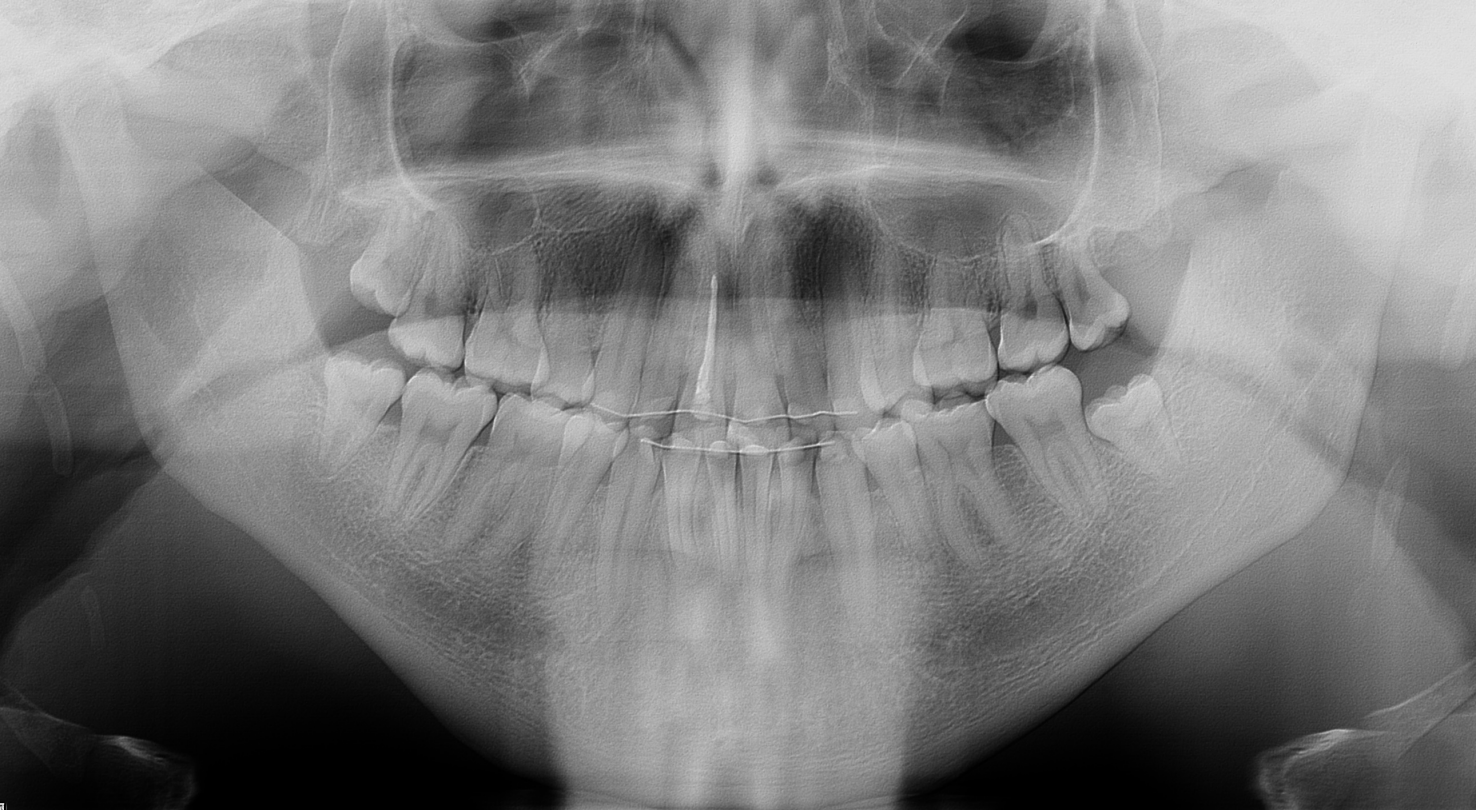
Postoperative orthopantomogram demonstrating successful removal of the coronoid processes.

**Fig. (8):**
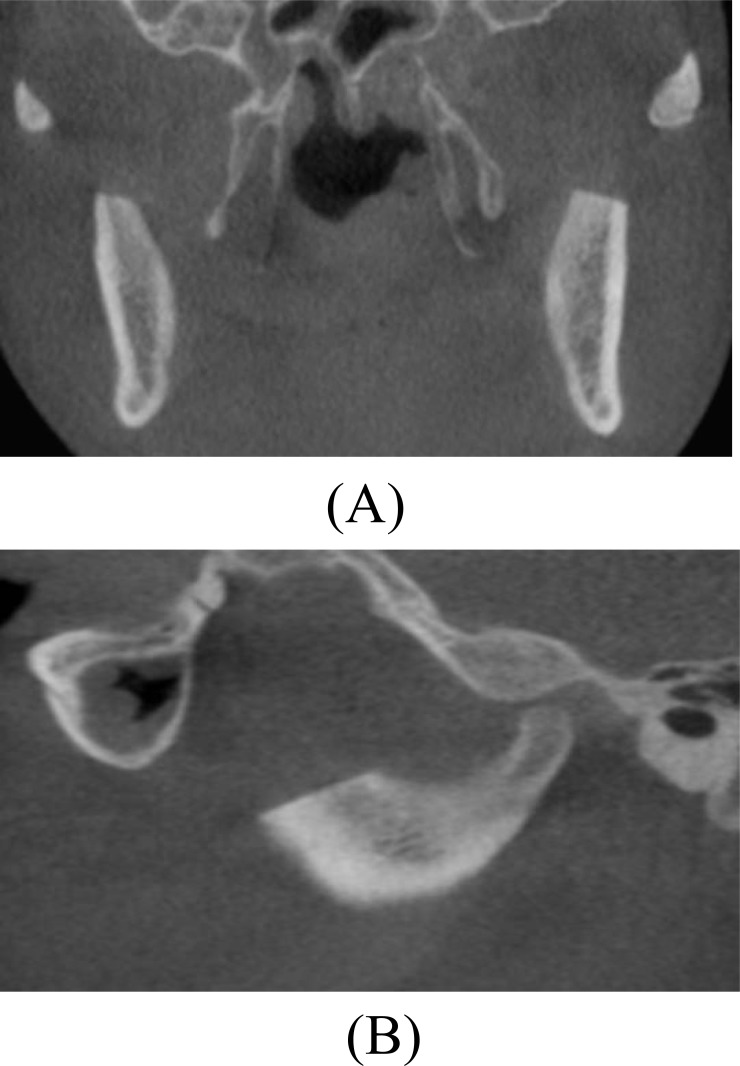
Postoperative Cone Beam Computed Tomography scan. (A) Coronal image showing no interference between the coronoid processes and the medial surface of the zygomatic bone and arch. (B) Sagittal image showing the resected coronoid process with no interference with the zygomatic bone.

**Fig. (9):**
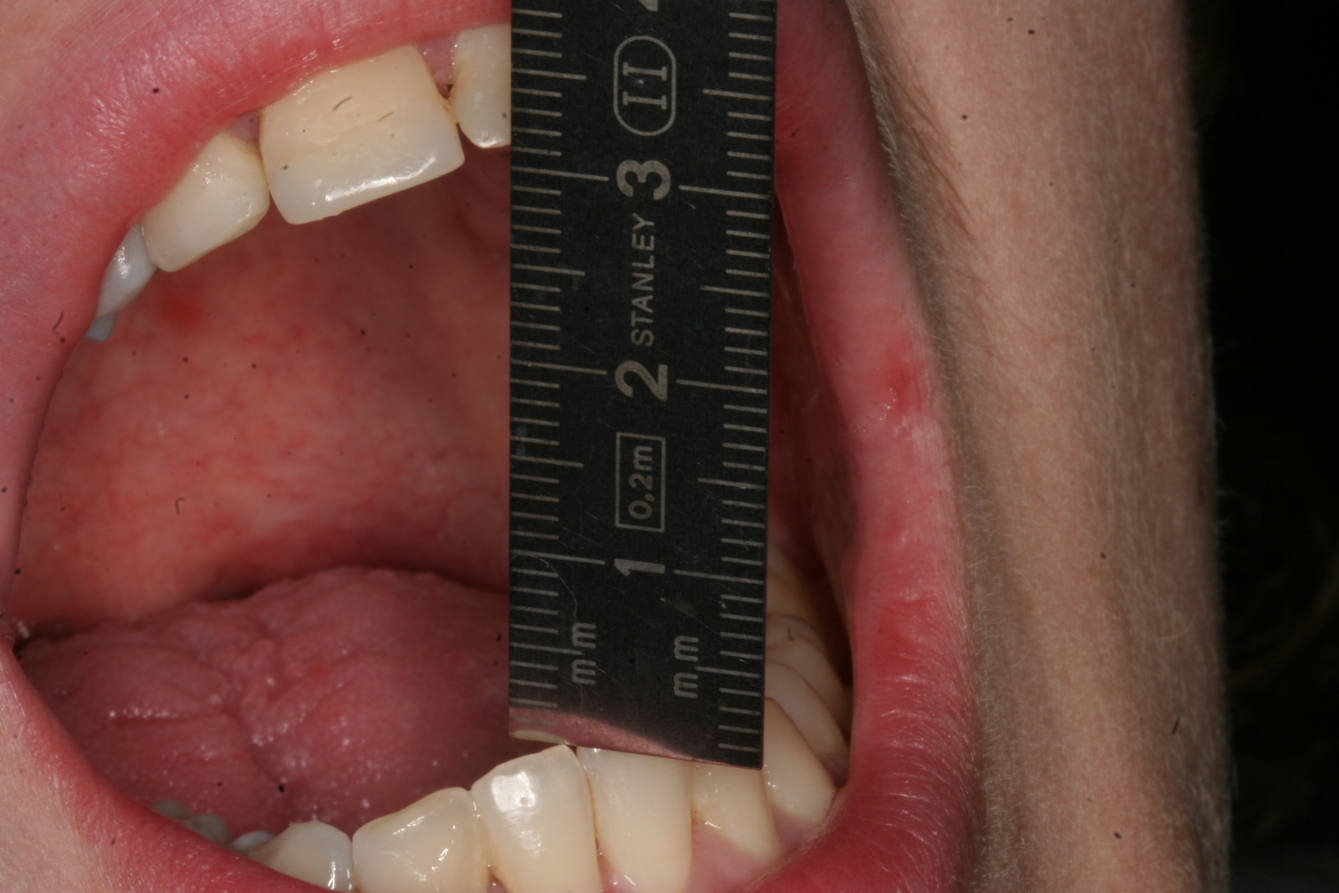
Three months postoperative maximal interincisal opening of 32 mm.

Post-operative panoramic and three-dimensional CT demonstrated successful bilateral coronoidectomy with no interferences between the mandible and zygomatic bone during mouth opening (Figs. **7** and **8**). Vigorous physical therapy was initiated one week after surgery using TheraBite Jaw Motion Rehabilitation System. However, the patient was not particularly motivated for physiotherapy due to pain and a tightening sensation in the cheek. Three months after the operation, a satisfactory mouth opening of 32 mm was achieved (Fig. **9**).

## 3 RESULTS

We present an 18-years old male with MCPH and limited mouth opening and summarize the current knowledge about this uncommon congenital or developmental temporomandibular joint disorder. The etiology of MCPH has not been established, although several theories have been suggested [1]. MCPH usually affects males in the second decade of life and the main clinical finding is a progressive painless reduction in mouth opening owing to the interference between the elongated coronoid process and the medial surface of the zygomatic bone [1].

Mouth opening limitations can be caused by a variety of factors including anterior disc displacement without reduction, masticatory muscle disorders, or temporomandibular joint osteoarthritis. The frequency of restricted mouth opening due to elongation of the coronoid process is rare. A previous study assessing 163 patients with limited mouth opening revealed that elongation of the coronoid process was found to be the cause in 5% of the patients [15].

Limited mouth opening due to MCPH is frequently misdiagnosed, since the condition is rare and not considered in clinical and radiographic examinations. Various diagnostic modalities have been suggested for patients with mouth opening limitation caused by MCPH [14]. Computed tomography with three-dimensional reconstruction is the best imaging modality for accurate visualising of MCPH interfering with the medial surface of the zygomatic arch, as it provides a detailed anatomy of the elongated coronoid process and the relationship with adjacent structures, revealing the points where impaction occurs on the zygomatic bone. Moreover, three-dimensional image reconstruction also contributes to the surgical planning.

Surgery is the only option to treat a mechanical obstacle involving MCPH with impingement on the zygomatic bone. MCPH with limited mouth opening has previously been treated with either coronoidotomy or coronoidectomy [1]. Coronoidotomy involves surgical detachment of the coronoid process from the mandibular ramus and has been used to treat several conditions, including coronoid hyperplasia and temporomandibular joint ankylosis [16]. The stability of the outcome, however, is considered questionable because of the risk of reattachment of the coronoid process [16]. However, coronoidotomy in combination with prolonged postoperative physiotherapy has demonstrated satisfying long-term results with substantial improvement in mouth opening [6, 17]. Coronoidectomy is the most commonly used surgical method for MCPH involving surgical excision of the elongated coronoid process [18]. Several studies have reported enhanced interincisal mouth opening after coronoidectomy [1]. Coronoidotomy or coronoidectomy can be accomplished using intraoral and extraoral approaches. The intraoral approach is the most commonly used surgical method and has the advantages of providing sufficient access without producing any extraoral scar and morbidity to the facial nerve, but the ease of accessibility is limited. However, the intraoral approach is associated with risk of haematoma and subsequently fibrosis. Endoscopically assisted intraoral coronoidectomy has therefore been advocated due to direct vision, no extraoral approach, easy and safe osteotomy with minimal risk of damage to the medial tissue [18]. The decision regarding the type of surgical technique and approach should depend upon the visibility, risk of complications and cosmetic patient demands. Consequently, the surgical approach to remove an elongated coronoid process should be case specific and the intraoral approach is preferable when possible.

Postoperative physical therapy is very important for obtaining a good result after coronoidectomy [9, 14]. Various physiotherapeutic techniques and strategies have been suggested involving spatula, wedge, and TheraBite Jaw Motion Rehabilitation System [3, 9, 14]. The exercise program should be initiated shortly after surgery and the patients should be instructed to perform the exercises several times a day involving maximum mouth opening and translation movements in all directions.

Mandibular coronoid process enlargement can result from exostosis, osteoma, osteochondroma, chondroma, hyperplasia and developmental anomalies [19]. Enlargement of the mandibular coronoid process can also be related to Jacob´s disease, which is a rare condition characterized by formation of a pseudo-joint between the enlarged mandibular coronoid process and the inner aspect of the zygomatic arch [19]. Jacob´s disease is usually seen in young males and the most common clinical presentation is a progressive limitation in mouth opening [19]. The etiology and pathogenesis of Jacob´s disease is unknown and the pseudo-joint can be treated successfully with coronoidectomy [19].

## 4 DISCUSSION

In this case report, bilateral coronoidectomy and postoperative physical therapy were performed in an 18-years old male with restricted mouth opening due to mandibular coronoid process impingement. The interincisal mouth opening increased from 22 mm to 32 mm, 3 months after surgery. Progressive limitation in mouth opening is a common clinical finding of mandibular coronoid process impingement. The present case report enhances the current knowledge about diagnosing and treatment of this uncommon congenital or developmental temporomandibular joint disorder.

## CONCLUSION

An 18-years old male with MCPH and limited mouth opening has been presented and the current knowledge about this uncommon congenital or developmental temporomandibular joint disorder has been discussed. The clinical and radiographic appearance of MCPH is very characteristic, and elongated coronoid process impingement on the medial surface of the zygomatic arch is a well-known cause for limited mouth opening. Therefore, the association of MPCH and limited mouth opening must always be kept in mind and General Dental Practitioners must have knowledge of this congenital or developmental temporomandibular joint disorder.
